# Supplementary material for: The interactome of intact mitochondria by cross-linking mass spectrometry provides evidence for coexisting respiratory supercomplexes
Source: Mol Cell Proteomics. 2017 Dec 8;17(2):216–32. doi: 10.1074/mcp.RA117.000470 (PMC5795388; doi:10.1074/mcp.RA117.000470)
Supplement: Supplemental Data [file supp_RA117.000470_133922_0_supp_23978_fzffwf.zip › spectra_annotation/mito_DR_spectra_annotation/310-1-7-1-5-1.pdf]

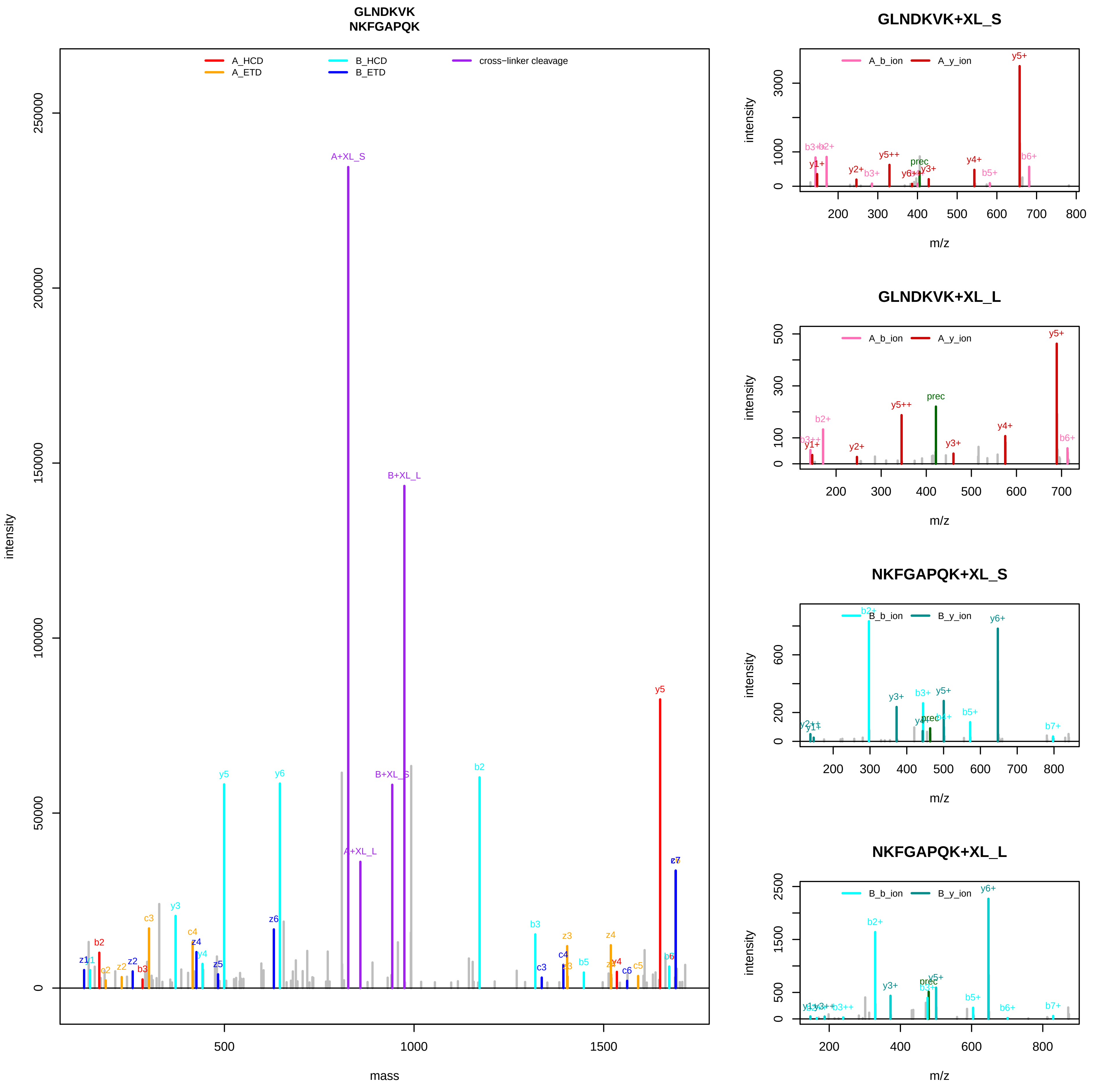
GLNDKVK
NKFGAPQK
A_HCD
A_ETD
B_HCD
B_ETD
cross−linker cleavage
A+XL_S
B+XL_L
B+XL_S
A+XL_L
y5
y6
b2
y3
c3
c4
z4
y4
z6
b3
z3
z4
y4
c5
z1
b2
z2
b3
z5
c3
c4
b5
c6
b6
c7
z7
intensity
mass
0
50000
100000
150000
200000
250000
500
1000
1500
GLNDKVK+XL_S
A_b_ion
A_y_ion
y5+
y4+
b6+
b5+
y5++
y2+
b3+
y3+
prec
y1+
b3++
b2+
y6++
GLNDKVK+XL_L
A_b_ion
A_y_ion
y5+
prec
y5++
b2+
y4+
b6+
y3+
y2+
y1+
b3++
NKFGAPQK+XL_S
B_b_ion
B_y_ion
b2+
y6+
y3+
b3+
y5+
b5+
b7+
prec
b4+
y4+
y2++
y1+
NKFGAPQK+XL_L
B_b_ion
B_y_ion
y6+
b2+
y3+
prec
y5+
b3+
b5+
b6+
b7+
b3++
y1+
y3++
intensity
m/z
